# Use and traditional management of *Anadenanthera colubrina *(Vell.) Brenan in the semi-arid region of northeastern Brazil

**DOI:** 10.1186/1746-4269-2-6

**Published:** 2006-01-18

**Authors:** Júlio Marcelino Monteiro, Cecília de Fátima CB Rangel de Almeida, Ulysses Paulino de Albuquerque, Reinaldo Farias Paiva de Lucena, Alissandra Trajano N Florentino, Rodrigo Leonardo C de Oliveira

**Affiliations:** 1 Departamento de Biologia, Área de Botânica, Laboratório de Etnobotânica Aplicada, Universidade Federal Rural de Pernambuco, Rua Dom Manoel de Medeiros s/n, Dois Irmãos, Recife, Pernambuco, 52171-030, Brazil

## Abstract

The use and management of "angico" (*Anadenanthera colubrina *(Vell.) Brenan) by a rural community in northeastern Brazil was examined. By employing different techniques of data collection and population structure analysis, it was determined that this species had multiple uses within the local community (especially as timber and for other wood products), and that local management of this species is based on simple maintenance and harvesting of individuals in agroforest homegardens. The study of the population structure of this tree species indicated that management and conservation strategies must include the participation of the local community.

## Introduction

The *Caatinga *ecosystem covers approximately 800,000 km^2 ^of northeastern Brazil. This region is marked by a severe climate with accentuated dryness (rainfall is usually less than 600 mm/yr) [1]. This ecosystem has suffered from the effects of increasing anthropogenic alteration for many decades, resulting in the conversion of extensive areas to pasture or farm land and the intensive harvesting of wood products, especially as energy sources [2-7].

Woody *caatinga *species are employed for many different purposes, especially the direct use of wood products. *Anadenanthera colubrina *(Vell.) Brenan, known in northeastern Brazil as "angico", for example, is largely used in rural constructions, as an energy source, as well as in popular medicines [2,3]. In light of its ample use, "angico" was indicated as high priority for *in situ *conservation at the 1^st ^Technical Reunion for "Strategies for the Conservation and Management of the Genetic Resources of Medicinal and Aromatic Plants in Brazil" [8].

**Figure 1:**
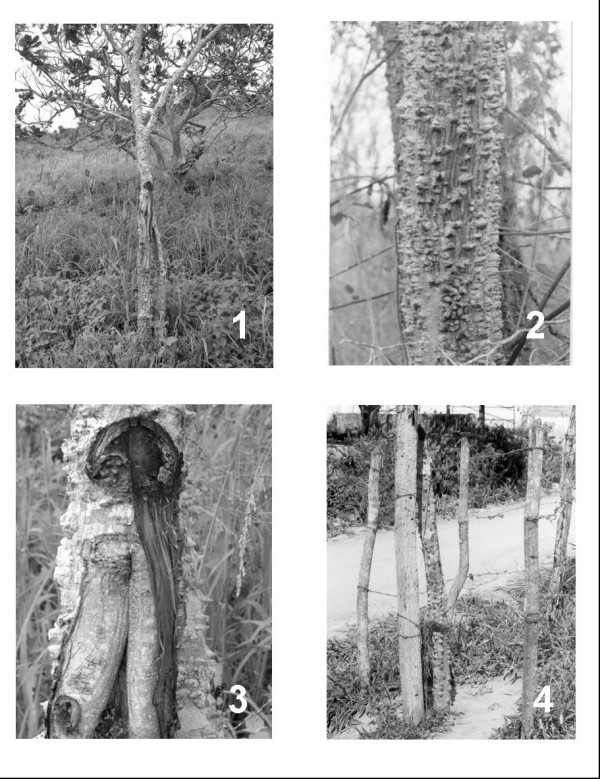
*Anadenanthera colubrina *(Vell.) Brenan. Caption: 1. General aspect of the species; 2. Detail of the trunk bark; 3. Detail of the bark during regeneration; 4. Use for fences.

"Angico" belongs to the family Mimosaceae and is widely distributed in the *caatinga*. The tree grows to between 5 and 20 meters tall, and the trunk has large numbers of conspicuous thorns (characteristic of this species) [9] (Figure 1). It is used in traditional medicine to treat respiratory problems and inflammations, as well as in industry for tanning leather [10]. The seeds of *Anadenanthera peregrina *(L.) Speng., an related species, are used to prepare *yopo*, a hallucinogenic inhalant used by the *curandeiros *of the *Piaroa *tribe inhabiting southeastern Venezuela [11]. The use of *yopo *as a hallucinogenic among indigenous peoples of Latina America has been confirmed by archeological evidences [12].

The present work examines the use of *A. colubrina *by a rural community in the semi-arid region of Pernambuco State in northeastern Brazil. We also attempted to identify the techniques employed in the local management of this species, and to evaluate the sustainability of this use by examining the population structure of the tree populations.

## Materials and methods

### Study site

The study described here was undertaken in the community of Riachão de Malhada de Pedra, situated near the "Empresa Pernambucana de Pesquisa Agropecuária (IPA)" experimental station in the municipality of Caruaru, Pernambuco State (8°14'19" S, 35°55'17" W), approximately 136 km from the state capital in Recife, Northeastern Brazil. The area has a hot, semi-arid climate, with an average temperature of 22°C, and an average yearly rainfall of approximately 609 mm concentrated within the months of June and July [13]. The families Euphorbiaceae, Mimosaceae, Fabaceae, Asteraceae, and Myrtaceae are well represented in the local vegetation, and many of their species are of great importance to the local community, such as *Caesalpinia pyramidalis *Tul.*, Myracrodruon urundeuva *(Engl.) Fr. All., and *Anadenanthera colubrina *(Vell.) Brenan [14].

### Ethnobotanical approaches

Ethnobotanical data was collected by means of semi-structured interviews undertaken during the period from January 2003 until July 2004. Visits were made to 98 of the 117 residences in the community, and 55 men and 43 women were interviewed. All of the interviewees were family heads, independent of their sex or age, and were older than 18. All interviews were voluntarily and consisted of basic questions concerning local use of the species under investigation. Gender related differences in knowledge were tested using the chi-square test [15].

Principal informants (19) were chosen, among all the interviewees, in order to complement and test information furnished by the other interviewees. These principal informants were selected based on indications by the local residents themselves and on the quality of the information they furnished during the interviews. These informants included people with a vast knowledge of the local native flora [16]. Guided tours were undertaken at the same time as the interviews. This technique consists of visits to the field, accompanied by some of the principal informants that made themselves available, in order to examine the plants 'in loco', and to collect botanical material for scientific identification [17].

Interviews, with all of the interviewees, were also based on the "checklist/inventory" [18,17], which consists of showing the interviewees photographs of the species under study. Additionally, an interview form was filled out containing the information (local knowledge) concerning a given species. Interviewees were first queried to ascertain if they were familiar with the plant in question, and if the answer was affirmative, the questioning would proceed. Direct observation consisted of accompanying the daily activities of the local inhabitants, while avoiding any direct interference with their actions. This technique allowed us to elucidate some facts that were left unclear after the interviews themselves [17]. A number of excursions were made within the community itself during the phase of direct observation in order to observe and note the local activities and practices involving the species under study. The objective of these observations was to obtain auxiliary information that might not have appeared during the interviews.

The interviews were recorded using small tape recorders, with the overt consent of the interviewees. After taping, the interviews were transcribed and the information transferred to a data bank. Plant material was identified through the use of analytical keys, through comparison with material deposited in the Vasconcelos Sobrinho Herbarium (PEUFR) at the Universidade Federal Rural de Pernambuco, as well as by consulting specialists. Voucher specimens were incorporated into the PEUFR herbarium (numbers 46644 to 46653).

### Management and local availability of *Anadenanthera colubrina* (Vell.) Brenan

In order to characterize the population structure of *A. colubrina*, 50 contiguous, semi-permanent 10 × 10 meter plots were established in each of two distinct areas of the same forest fragment, totaling 100 plots, covering one hectare of land. One of the areas was located close to the community being studied, while the other plot was established approximately 2 kilometers more distant. This latter area was chosen, for comparative purposes, because it had been systematically monitored using semi-permanent plots for at least ten years in ecological studies. As such, the same procedures were extended to the area closest to the community. The inventories sampled all species present in the surveyed plots.

All individuals with a diameter at ground level ≥ 3 cm were surveyed, common procedure for studies in the *caatinga *dry-land vegetation [19]. The sample plot method, although more labor-intensive, allows the researcher to monitor the populations under study, accompany their phenological cycles, and collect material for botanical identification. Additionally, it is a method commonly used for studying population structure. Measurements of height and diameter at ground level were recorded for each plant, as well as relative density and basal area (Mueller-Dumbois & Ellenberg [20]. Estimates of tree height were performed using a standardized reference. All analyses were based on live and entire individuals, or plants that had died through apparently natural processes.

The numbers of individuals showing signs of having been selectively cut were also noted. Primary information concerning the management of this species and the areas from which they were extracted were obtained from conversations with the interviewees and the principal informants. Differences in age-structures between the two populations were verified using the Kruskal-Wallis test [21].

## Results and Discussion

### Local uses

Informants indicated a number of different uses for the tree species *A. colubrina*, including: construction, medicinal use, fuel, artifacts, and forage. The use of *A. colubrina *as a source of forage was indicated by one informant who commented on the use of the leaves to feed cattle. However, Tokarnia et al. [22] have indicated that the leaves of this tree contain large quantities of cyanogenic glycosides capable of causing cyanic intoxication. It is apparently well known among residents of rural areas that the leaves of this species are quite toxic to cattle.

The use of the wood of *A. colubrina *for making artifacts was reported in 0.8% of the citations. These uses included the confection of simple smoking pipes or ornamental pieces such as pencil-holders. These observations demonstrated that the inhabitants of the Riachão village have a wide knowledge of the uses of wood from this species. Only one informant from the community sold his handicrafts at local markets, and only on a modest scale.

The use of this species for fuel was indicated by 32.3% of the citations (directly related to household cooking). The informants noted that this species was preferred over other local plants for cooking as it produced intense and long-lived flames. One of the informants, however, indicated that this wood was also used by a local bakery (Table 1). The use of plants as energy sources is very common in rural communities in arid and semi-arid regions [23,24]. In Nigeria, 80% of the energy for cooking is collected from forest resources, corresponding to 43.4 × 10^9 ^kg [23]. In many regions there are no available alternatives to wood [23]. This as true in the community studied here, where wood is used almost exclusively in cooking due to the high costs of natural gas.

**Table 1:** Number of citations of the use of *Anadenanthera colubrina *(Vell.) Brenan, in the rural community of "Riachão de Malhada de Pedra", municipality of Caruaru (Pernambuco, Northeastern Brazil). Number of informants = 98.

Categories	Sub-categories	Number of citations
**Fuel**	Charcoal	49
	Firewood	29
**Medicine**		83
**Construction**	Fence	71
	Line timber	04
	Roof timber	01
	Board timber	01
**Technology**	Handicraft	02
**Fodder**		01

The construction category concentrated 32% of the citations, and 92% of the uses mentioned referred to the construction of fences (Table 1). The construction of fences using native *caatinga *plants is an extremely common practice in the whole of northeastern Brazil, and *A. colubrine *was highly preferred in the study region. Preference for this tree species is due to its durability and resistance to termites. Fence posts of this species can last about five years, according to informants. Fence repair is sporadic, and posts that are replaced are subsequently used as firewood. This same species was also used in house construction, but this practice has been largely abandoned due to the difficulty in finding plants of the right size and form. Members of the local communities have taken to buying exotic species from lumberyards for building.

The use of *A. colubrina *in folk medicine is very common, comprising 34.4% of all citations. The bark (80 citations) is most commonly used in preparing these medicines. The leaves and inner bark were cited only twice, while the fruits were cited but once. The excessive harvesting of bark from could result in serious problems for the populations of the species as intensive and indiscriminant collecting could kill the plants. Zschocke et al. [25] pointed out that the leaves (or other plant tissues) could possibly substitute for the bark used in many medicines. Such a substitution, however, would require phytochemical and pharmacological studies to evaluate their security and effectiveness. In the *caatinga*, however, leaves are only sporadically available as their production is dependent on the ephemeral rains. Bark, on the other hand, is always available [2]. A sugary drink (*syrup*) is prepared locally from the bark (48% of the total citations). Other preparations cited (46%) include decoction, tinctures, "garrafadas" (infusion of many plants in alcohol), and teas that are used to treat a number of ailments.

### Distribution of knowledge

Men and women apparently retain different forms of knowledge concerning the use of these species. The men tend to detain more information concerning the use of woody species, while women tend to specialize in plants that do not have wood-uses, especially among the medicinal plants [26,27]. In the present study, women demonstrated greater knowledge of the therapeutic use of *A. colubrina *than men did, supplying 54% of the responses in this category. The reverse was seen, however, within the categories of fuel-use and construction, where the largest numbers of citations were supplied by men (54% and 55%, respectively). These differences were not, however, statistically significant (x^2 ^= 2.34; p > 0.05).

Although some researchers have affirmed that no significant gender related distinctions related to the knowledge of medicinal species have been observed [28], others have demonstrated such distinctions and related them to cultural factors. Men tend to have a better knowledge of woody resources as they tend to spend more time in forest environments, while women usually show greater familiarity with subjects related to domestic attributes, as they tend to spend more time in the home garden [26,27]. Marshall and Newton [29], for example, found that the collection and knowledge of non-woody forest products were primarily female activities (with children regularly involved). Figueredo et al. [30] also found evidence of gender-specific knowledge, as women tended to recognize (and manipulate with greater frequency) plants considered to be medicinal. These observations suggest that the effectiveness of projects involving the development, management and conservation of natural resources will hinge on taking into consideration which sector of the population retains the principal knowledge and use of those resources.

### Management and local availability

There are no especially designated zones for wood extraction in the region examined, nor extraction patterns that could be identified during walks in the forest fragment or during informal conversations with the local population. In agreement with both the informants and field observation, wood products used in construction are obtained in the areas closest to the community, and are collected during excursions with that specific purpose. Individuals of *A. colubrina *that are used for medicinal purposes or shade are occasionally preserved in larger homegardens. As such, there appear to be only two degrees of attention given to this species: collection and tolerance. When preparing new areas for cultivation, this species is normally harvested for rural construction. Most of the studies concerning incipient management in Central American regions have focused on edible plants [31-33]. The management techniques identified in the community under study here are consistent with patterns observed in other Neotropical regions [34], although close comparisons with these works are more difficult as *A. colubrina *is essentially managed only for wood-use.

**Figure 2:**
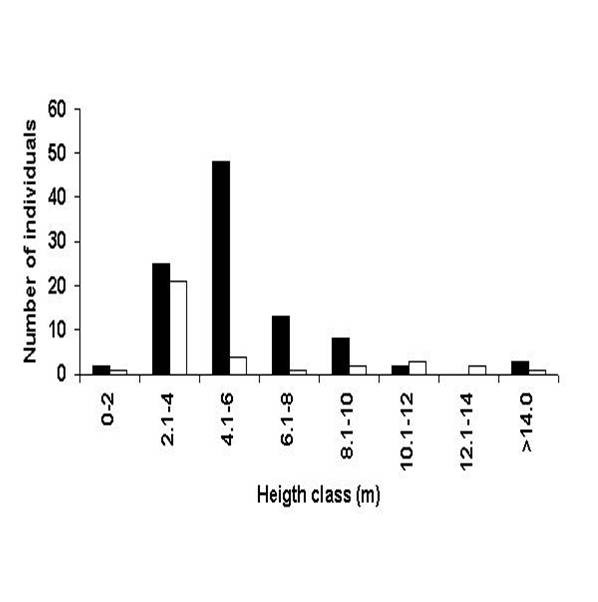
Distribution of individuals of *Anadenanthera colubrina *(Vell.) Brenan according to height class in the two study areas, municipality of Caruaru (Pernambuco, Northeastern Brazil). Area_1 _■ = more distant study site; Area_2 _□ = closer study site.

**Figure 3:**
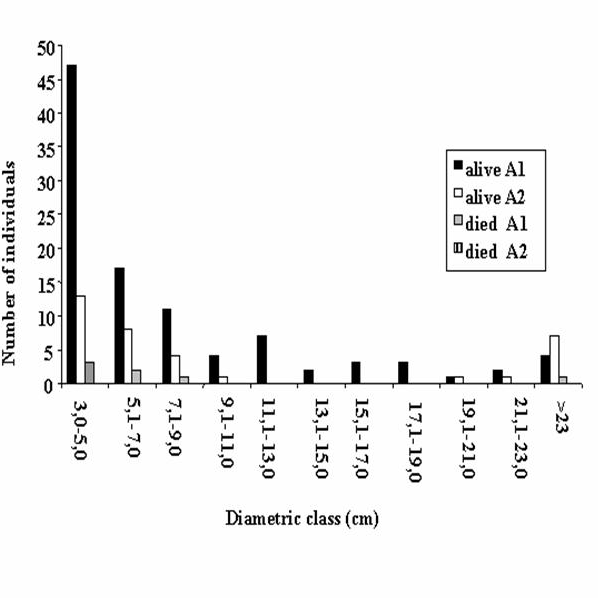
Distribution of the individuals of *Anadenanthera colubrina *(Vell.) Brenan in diametric class in two fragmented areas of *Caatinga*, municipality of Caruaru (Pernambuco, Northeastern Brazil). Area_1 _■ = more distant study site; Area_2 _□ = closer study site.

The analysis of the population structure of *A. colubrina *was undertaken in order to examine the local availability of this species, to estimate its degree of exploitation, and to determine if these factors where related to the proximity of the forest fragment to the community. The distribution of the number of individuals by height reveals that the areas furthest (A_1_) from the community had large numbers of individuals in the smallest height classes, but few individuals in the tallest classes (Figure 2). In the areas closest (A_2_) to the community, there were only small numbers of individuals in all of the height classes. The numbers of dead individuals was very low in both areas (Figure 3). The survival rate of the seedlings of this species after the first rains is quite high (73.3%) [9], as they are resistant to environmental stress, demonstrating survival mechanism such as leaf loss and the reduction of metabolism during long periods of water-stress [9].

An analysis of the diameter classes revealed that the numbers of individuals are distributed according to the "Inverted J" model in the area most distant from the community, with the largest numbers of individuals being found in the smallest diameter classes. Luken [35] suggests that this type of distribution indicates an equilibrium situation between germination and death. In the area nearest the community the greatest concentration of individuals is also found within the smallest size classes, but there was, however, a marked absence of individuals in the intermediate diameter classes, exactly those classes preferred for fence posts and firewood (Figure 2). Francelino et al. [36] observed that trees with diameters between 7 and 14 cm are potentially useful for construction work, and virtually all individuals = 2 cm can be used for firewood. Although the persistence of at least some individuals in the largest diameter classes would appear to favor the local continuity of this species, continued extraction may threaten these populations. Similar situations been seen in other localities, where tree populations are dominated basically by young individuals [9,36].

The structures of the close and distant populations (Table 2) studied here differ significantly in terms of height classes (A_1_= 5.67 m ± 2.61 m; A_2_= 5.64 m ± 3.96 m), but not in relation to diameter classes (A_1_= 8.6 cm ± 8.6 cm; A_2_= 11.50 cm ± 12.30 cm) and basal area (A_1_= 0.11 m^2 ^± 0.32 m^2^; A_2_= 0.22 m^2 ^± 0.46 m^2^) according to the Kruskal-Wallis Test at a 5% level of probability. The differences observed in relation to height can possibly be explained by habitat conditions. The forest fragment grows on slightly hilly land, while the forest more distant from the community occupies more humid lowlands near a river. Interestingly, no significant differences were noted between the two populations in terms of their basal area or their diameters. The trees in the area nearest the community, however, had distinctly smaller populations, with a marked reduction in individuals within the diameter classes preferred for local use.

**Table 2:** Population structure of *Anadenanthera colubrina *(Vell.) Brenan, in the rural community of "Riachão de Malhada de Pedra", municipality of Caruaru (Pernambuco, Northeastern Brazil. A1 = more distant study site; A2 = closer study site. The different letters indicate significant differences in the Kruskal-Wallis test (p < 0,05). X = Average, SD = Standard Deviation

**Area**	**No. of Individuals**	**Relative Density**	**Average height (m) X **± SD	**Diameter (cm) X **± SD	**Basal area (m**^**2**^**) X **± SD
A1	101	4.9	5.67 ± 2.61^a^	8.6 ± 8.6^a^	0.11 ± 0.32
A2	35	1.9	5.64 ± 3.96^b^	11.50 ± 12.30^a^	0.22 ± 0.46

## Conclusion

*A. colubrina *is an important species to the communities in the region examined, and it has a good number of categories of uses, especially in terms of construction and home remedies. The population structure of this species indicates that it should receive special attention to develop management and conservation strategies, and that the programs must be undertaken in conjunction with the local community. One alternative to diminish the use-pressure on this species would be to promote the local cultivation of these trees, diminishing pressure on the remaining native populations. Social research can help in developing strategies for the management and sustainable use of this species.
